# Comprehensive functional characterization of *SGCB* coding variants predicts pathogenicity in limb-girdle muscular dystrophy type R4/2E

**DOI:** 10.1172/JCI168156

**Published:** 2023-06-15

**Authors:** Chengcheng Li, Jackson Wilborn, Sara Pittman, Jil Daw, Jorge Alonso-Pérez, Jordi Díaz-Manera, Conrad C. Weihl, Gabe Haller

**Affiliations:** 1 Department of Neurology and; 2 Department of Neurosurgery, Washington University School of Medicine, St. Louis, Missouri, USA.; 3 Neuromuscular Disease Unit, Neurology Department, Hospital Universitario Nuestra Señora de Candelaria, Fundación Canaria Instituto de Investigación Sanitaria de Canarias, Tenerife, Spain.; 4 John Walton Muscular Dystrophy Research Center, Newcastle University, Newcastle Upon Tyne, United Kingdom.; 5 Department of Genetics, Washington University School of Medicine, St. Louis, Missouri, USA.

**Keywords:** Genetics, Muscle Biology, Genetic variation, Neuromuscular disease

## Abstract

Genetic testing is essential for patients with a suspected hereditary myopathy. More than 50% of patients clinically diagnosed with a myopathy carry a variant of unknown significance in a myopathy gene, often leaving them without a genetic diagnosis. Limb-girdle muscular dystrophy (LGMD) type R4/2E is caused by mutations in β-sarcoglycan (*SGCB*). Together, β-, α-, γ-, and δ-sarcoglycan form a 4-protein transmembrane complex (SGC) that localizes to the sarcolemma. Biallelic loss-of-function mutations in any subunit can lead to LGMD. To provide functional evidence for the pathogenicity of missense variants, we performed deep mutational scanning of SGCB and assessed SGC cell surface localization for all 6,340 possible amino acid changes. Variant functional scores were bimodally distributed and perfectly predicted pathogenicity of known variants. Variants with less severe functional scores more often appeared in patients with slower disease progression, implying a relationship between variant function and disease severity. Amino acid positions intolerant to variation mapped to points of predicted SGC interactions, validated in silico structural models, and enabled accurate prediction of pathogenic variants in other SGC genes. These results will be useful for clinical interpretation of *SGCB* variants and improving diagnosis of LGMD; we hope they enable wider use of potentially life-saving gene therapy.

## Graphical abstract

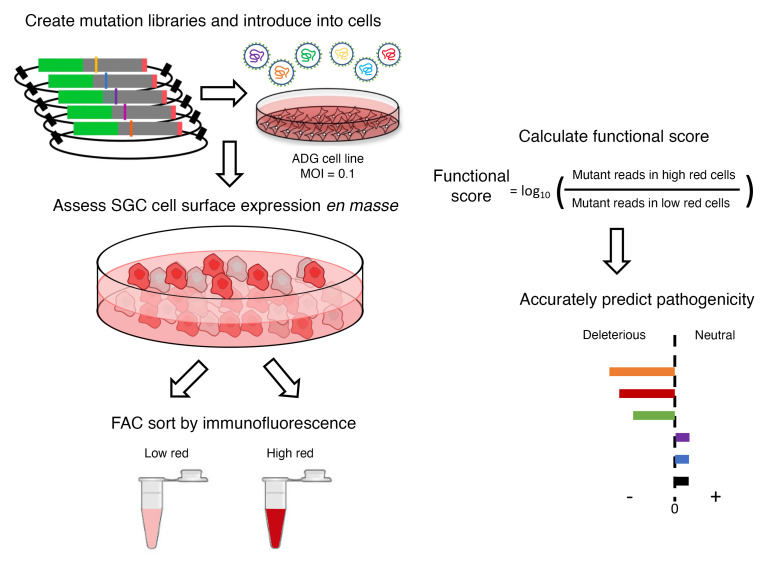

## Introduction

Recessive mutations in β-sarcoglycan (*SGCB*) cause limb-girdle muscular dystrophy type R4/2E (LGMDR4/2E), resulting in muscle wasting, progressive weakness, degeneration of skeletal muscle, and often premature death (1, 2). β-Sarcoglycan is a key component of the dystrophin-associated protein complex (3–5). In muscle cells, the dystrophin-associated protein complex localizes to the membrane and connects the intracellular cytoskeleton to the extracellular matrix, allowing for coordinated force production in muscle. The dystrophin complex also acts as a membrane stabilizer during muscle contraction to prevent contraction-induced damage (6, 7). The sarcoglycan subcomplex is composed of 4 single-pass transmembrane proteins: α-sarcoglycan, β-sarcoglycan, γ-sarcoglycan, and δ-sarcoglycan (8). The sarcoglycan subunits assemble and translocate within the myofiber as a complex, and loss of any individual subunit due to loss-of-function mutations adversely affects the stability and trafficking of the unmutated sarcoglycan proteins, leading to what is referred to as sarcoglycanopathy (9–11). A handful of missense pathogenic variants have been identified in each sarcoglycan (12–14). These missense mutations lead to a failure in sarcolemmal localization and sarcoglycan complex formation (14, 15). Currently, no crystal or cryo-EM structure exists for this essential membrane complex that is critical to human health. Thus, whether missense mutations in sarcoglycans destabilize the protein, alter its trafficking to the sarcolemma, or affect its interactions with its sarcoglycan partners is not fully understood.

Clinical diagnosis of sarcoglycan-deficient LGMD currently requires histopathologic assessment of a patient’s muscle biopsy for cell surface–localized sarcoglycan complex proteins or biochemical assessment of the protein’s presence (9, 10). Loss of one sarcoglycan subunit often secondarily leads to disruption of the entire sarcoglycan complex, further confounding a true diagnosis without genetic confirmation (15). Because of this apparent overlap in phenotype between the sarcoglycanopathies, and the phenotypic heterogeneity of other genetically defined LGMDs, obtaining a genetic diagnosis can be challenging. Genotype-phenotype correlations are emerging within the sarcoglycanopathies. For example, some mutations in SGCB are associated with late disease onset in the second decade of life, whereas other mutations result in early adolescent onset (13, 14). Moreover, the challenge of diagnosing patients with LGMDR4/2E before symptom onset or early in the course of the disease has the potential to enable the use of preventative gene therapy or other therapeutics, making the disorder highly clinically actionable.

Missense changes constitute the majority of variants observed in patients with LGMD, and, in most instances, particularly for recessive conditions, there is insufficient evidence to classify variants as pathogenic or benign, resulting in the designation as a variant of unknown significance (VUS) (14, 16, 17). VUSs present a diagnostic dilemma to patients and clinicians. At present there is no systemized path forward for “variant resolution.” The American College of Medical Genetics and Genomics (ACMG) has proposed strict criteria to assert the pathogenicity of a disease variant. One underutilized criterion in LGMD genes is PS3 (strong evidence, i.e. high accuracy in defining pathogenicity). The use of PS3 requires that a variant be assessed using a well-established in vitro or in vivo functional study to support a damaging effect on the gene or gene product. This process requires the development of gene-specific functional assays, making an individualized single patient variant resolution pipeline labor intensive and prohibitively expensive. To address this current gap in variant resolution and assess the function effect of LGMD missense variants, we employed deep mutational scanning (DMS) to measure the effects of all possible missense variants of the *SGCB* gene.

## Results

### In vitro assay of SGCB variant function.

We established a human cell system to model SGCB variant function using engineered HEK293 cells. Testing for protein expression for the 4 sarcoglycan (SGC) proteins verified that HEK293 cells lacked detectable protein expression of any of SGC gene. Furthermore, expression of a SGCB fusion protein (YFP-SGCB-HA) alone led to minimal cell surface expression, as measured by immunofluorescence with an anti-HA antibody (Supplemental Figure 1; supplemental material available online with this article; https://doi.org/10.1172/JCI168156DS1). Coexpression with the other 3 untagged SGC proteins (SGCA, SGCG, and SGCD), however, led to robust cell surface expression of WT YFP-SGCB-HA. To create a stable HEK293 cell line capable of reliably transporting SGCB to the cell surface, we transduced HEK293 cells with a mixture of lentiviruses designed to express SGCA, SGCD, and SGCG. Single clones from these transduced cells were isolated and tested at multiple passages for expression of the 3 stably expressing SGC proteins (Supplemental Figure 2A). One clone that expressed each SGC protein at similar levels was chosen and deemed ADG-HEK cells; it was used for all subsequent experiments (Supplemental Figure 2B). To preliminarily validate the assay, we introduced single missense mutations into YFP-SGCB-HA that are predicted to affect the cellular expression of SGCB because they have been established previously as pathogenic (G167S, S114F) (8). In addition, we generated one missense mutation (Q11E) that has been reported as a VUS. Lentiviral expression of YFP-SGCB-HA-WT in ADG-HEK cells displayed strong cell surface expression in nearly all YFP-positive transduced cells using an HA antibody on unpermeabilized cells (Supplemental Figure 3). In contrast, ADG-HEK cells transduced with presumptive pathogenic variants had a significant decrease in cell surface expression of SGCB, as visualized by immunofluorescence of unpermeabilized cells (Supplemental Figure 4 and Supplemental Figure 5). In contrast, the VUS YFP-SGCB-HA-Q11E had normal cell expression that was comparable to that of YFP-SGCB-HA-WT in ADG-HEK cells, as visualized by immunofluorescence of unpermeabilized cells, suggesting that this variant has no effect on its function. To create a high-throughput and quantitative assay for SGCB membrane expression, we performed flow cytometry on similar populations of cells as above. In addition, to explore whether an impairment in SGCB would lead to a secondary loss in SGCA cell surface expression, we immunostained ADG cells transfected with YFP-SGCB-HA-WT or mutation-containing plasmids and immunostained unpermeabilized cells with an antibody against the extracellular domain of SGCA. Consistent with a loss of SGCB cell surface expression, there was a secondary loss of extracellular SGCA cell surface immunofluorescence (Supplemental Figures 5 and 6).

### A functional effect map of SGCB missense variants.

**Figure 1:**
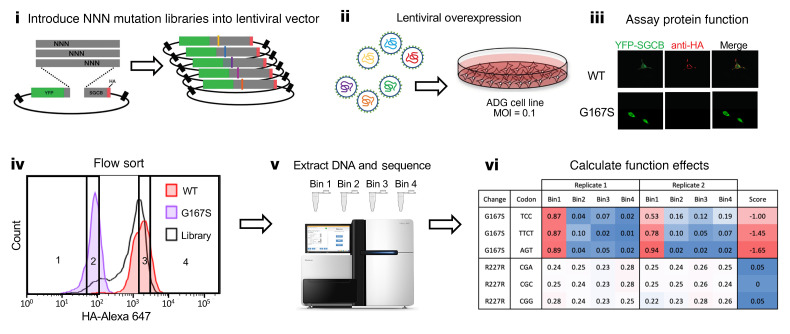
Overview of SGCB functional screen. Steps for generating and testing SGCB variant function in ADG-HEK human cells (stably expressing SGCA, SGCD, and SGCG). (i) Generation of mutation libraries by cloning synthesized pools of mutant oligos into a WT backbone. (ii) Creation and transduction of lentiviral libraries derived from the mutant plasmid libraries. (iii) Staining and imaging of transduced cells for YFP and HA antibody staining. Cells with pathogenic variants (G167S) fail to effectively transport intracellular SGCB (green) to the cell surface (red), while WT demonstrates robust total protein and cell surface expression of SGCB. (iv) Transduced cells sorted using FACS for HA staining into 4 bins. (v) Sequencing of cells with each bin of HA staining. (vi) Calculation of functional scores from mutation prevalence in each bin of HA staining. The resulting functional score is negative for deleterious variants or positive for functionally neutral variants.

**Figure 2:**
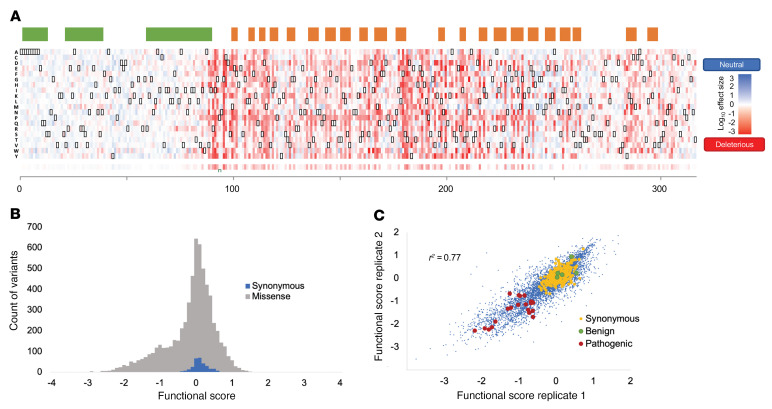
Functional effect map of SGCB. (**A**) α Helices (green) and β sheets (orange) as predicted from the AlphaFold2 multimer model of SGCB modeled with SGCA, SGCG, and SGCD. Functional score biological replicate median values for each amino acid change are displayed as a heatmap. Scores range from damaging (red) to benign variants (blue). Missing or low confidence data are shown in yellow. Synonymous changes are bounded in black boxes. Average HA functional score (constraint score) per position (318 amino acids) is shown below as a heatmap with each row being a different amino acid substitution labeled with the amino acid abbreviation (i.e., lysine = K). (**B**) Histogram of HA functional scores demonstrate a bimodal distribution with synonymous variants (blue) showing a narrow range of scores around 1 (i.e., enriched in HA bin 4). (**C**) Correlation among biological replicates of HA-stained ADG-HEK cells transduced with SGCB libraries (libraries A–F are included) and ClinVar pathogenic variants (red), benign variants (green), and synonymous variants (yellow) highlighted.

To test the effect of all possible missense *SGCB* variants, we used single amino acid saturation mutagenesis to generate libraries comprising every possible missense, synonymous, and nonsense variant. The *SGCB* cDNA (954 bp) was divided into 6 overlapping sublibraries (204–225 bp) to allow full-length sequencing of each. The mutant cDNA library for each sublibrary was cloned into the WT lentiviral YFP-SGCB-HA vector. ADG-HEK cells were transduced at low multiplicity (<0.1) to yield a population in which each cell expressed either 0 or 1 *SGCB* variant, recapitulating a hemizygous-like state in which to test the effect of each variant. Sequencing of both plasmid libraries and integrated libraries from genomic DNA demonstrated that nearly all single-codon mutations (98%) and nearly all amino acid changes (99%) were present and transduced into cells and the average depth of each mutation was generally uniform (range = 50–1,000 reads per million). Each cell population transduced with a SGCB mutant lentivirus pool was first bulk-sorted for expression of YFP to select cells expressing *SGCB*. YFP-positive cells were then grown for an additional 5–7 days and split into 2 equal populations and stained either for HA to detect YFP-SGCB-HA surface expression or with an antibody against the extracellular domain of SGCA to detect cell surface of SGCA. Cells were sorted into 4 bins corresponding to the top and bottom 10% of cells for either YFP-SGCB-HA or SGCA cell surface expression. To determine the relative abundance of each mutation in each bin, we performed amplicon sequencing of each mutated sublibrary. Functional scores for each variant were calculated for YFP-SGCB-HA cell surface expression (bin 1 vs. bin 4) and SGCA cell surface expression (bin 1 vs. bin 4) as the log_10_ ratio of the variant’s frequency in bin 4 (high expression) divided by its frequency in bin 1 (low expression), such that deleterious variants should score negatively and neutral variants positively (Figures 1 and 2 and Supplemental Figure 7). Functional scores ranged from –2.9 to 1.46, with all synonymous variants having a score of more than –0.5 (average = 0, range = –0.05 to 1.2) (Supplemental Table 2). In contrast, nonsense mutations had an average functional score of –1.5 ± 0.8. Using these values, we established cutoff scores for putative benign and pathogenic variants and normalized scores across chunks. Scores were not correlated with chunk of origin but did strongly reflect the protein domains and structural features predicted using AlphaFold2 (Figure 2A). Generally, positions before amino acid position 85 show a reduced likelihood of pathogenicity, and amino acid positions within the extracellular domains of the protein, particularly those within predicted β sheets showed an increased likelihood of pathogenic functional scores and scores demonstrated a bimodal distribution (Figure 2B). Functional scores were correlated across biological replicates (different transduced cell populations) (pairwise Pearson’s *r*^2^ = 0.77, Figure 2C).

### Functional scores validate existing variant interpretations.

**Figure 3:**
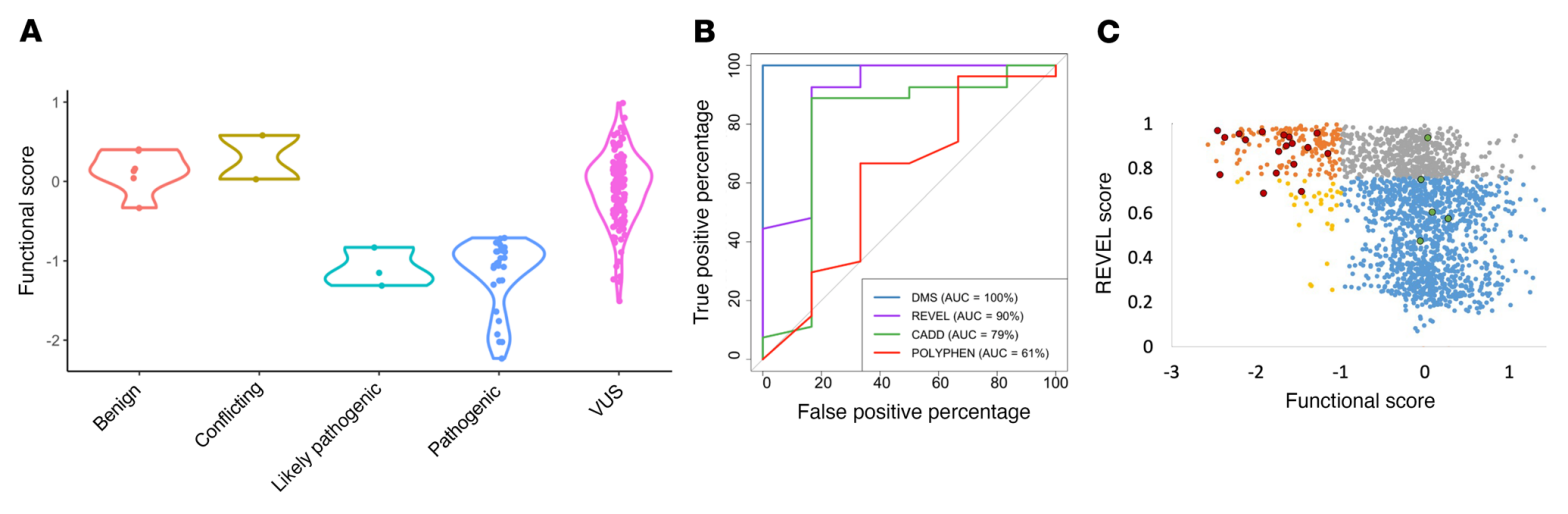
Concordance of functional scores with existing clinical classifications and computational predictions of pathogenicity. (**A**) Functional scores (*y* axis) for patient missense variants from ClinVar databases, by variant classification (*x* axis). (**B**) Receiver operator curves (ROC) predicting pathogenic or benign ClinVar classification (33 variants) for the functional score generated here (DMS), REVEL, CADD, or PolyPhen. (**C**) Concordance of computational predictor REVEL with DMS scores. Quadrants are distinguished by color scores of greater than or less than 0.75 and DMS scores of greater than or less than 0 and based on clinical classification as pathogenic/likely pathogenic (red) or benign/likely benign (green).

Having established cutoff scores, we found that pooled measurements recapitulated existing variant interpretations from ClinVar (18) and the Leiden databases (19) and recapitulated scores derived from our single variant experiments (Supplemental Figures 4 and 5). Notably, our functional scores agreed with the reported pathogenicity of all variants reported as pathogenic, likely pathogenic, benign or likely benign (Figure 3A), corresponding to classification sensitivity and specificity of 100%. These data provide evidence for the interpretation of the 99 VUSs in ClinVar or the Leiden SGCB databases. Using the functional scores derived here, we predict that 12 of these (12%) unresolved variants are functionally deleterious (Supplemental Table 3), a rate similar to the overall rate of nonfunctional variants possible from single nucleotide changes (283 of 2,250, 13%; *P >* 0.05, 2-sided binomial test). We also investigated the functional scores of variants present in healthy populations. We used these databases to test the false positive rate of our functional assay. Presuming that no one in these cohorts had LGMD, we expected that there should be no homozygotes with nonfunctional alleles by our assay and no compound heterozygotes with 2 nonfunctional alleles by our assay in any of these populations. First, we utilized the UK Biobank (UKBB; https://www.ukbiobank.ac.uk/) database. Among the UKBB population, there were 1,654 individuals with more than 0 nonsynonymous variants in *SGCB*. Of these, only 6 individuals harbored 2 nonsynonymous variants. In each instance, at least 1 of the 2 variants scored as functionally neutral/benign in our functional assay (Supplemental Table 4). We, therefore, predicted that none of the 488,248 patients present in the UKBB exome sequencing cohort had SGCB-deficient LGMD. Next, the gnomAD database (version 2.1.1; https://gnomad.broadinstitute.org/) lists 198 *SGCB* missense variants, all rare (maximum MAF, <0.2%). Of these variants, 20 variants (102 of 129,186, cMAF = 0.00079) were deleterious in our assay (score, <–0.5) and present in non-Finnish Europeans, with 0 of these observed as homozygotes. The most frequent variant that scored as pathogenic (S114F) is a known pathogenic variant in ClinVar and was observed in 68 non-Finnish European individuals in gnomAD, with none observed as homozygotes. Three *SGCB* missense variants were observed in the homozygous state in at least one population in gnomAD (R267C, F180L, and Y123S), but each scored as functionally neutral in our assay. These results reflect the mild negative selection on pathogenic variants in recessive disease genes that allows genetic drift to increase the frequency of even disease-causing variants below the level that would frequently produce homozygotes. Furthermore, the collective frequency of missense variants that we predicted were pathogenic in these 2 databases suggests a carrier frequency around 1 in 1,250–2,400 and, therefore, a population prevalence of SGCB-deficient LGMD of approximately 0.2–0.6 per million, ignoring the de novo mutation rate for this gene, which is consistent with various disease prevalence estimates in the literature (14, 17).

### Functional scores outperform bioinformatic predictors.

Bioinformatic tools are often used to aid in the interpretation of clinical variants. Methods are seldom protein specific and are based largely on evolutionary conservation, which for highly conserved genes often leads to inflated sensitivity but limited specificity in pathogenicity predictions. We compared the classification performance of our functional scores with those of 3 computational predictors: PolyPhen2, CADD, and REVEL (20–22). We used ClinVar pathogenic, ClinVar benign, and variants for which homozygotes have been observed in gnomAD or the UKBB (presumed benign) as a set of true positive and true negative variants. Measured functional scores were perfectly concordant with published pathogenicity assessments (Figure 3A). Functional scores outperformed each bioinformatic method in predicting pathogenicity, producing an AUC of 1; the next best predictor was REVEL, with an AUC of 0.9 (AUC range, 0.6–0.9; Figure 3B). However, the value of REVEL scores in predicting pathogenicity is likely inflated, owing to the lack of known benign variants with which to evaluate it. Given the large number of variants with functional scores of more than 0 (i.e., functionally benign) but damaging REVEL scores (>0.75; 537 of 1,870; 29; Figure 3C), it remains possible that the false positive rate for REVEL scores (i.e., benign variants called pathogenic by REVEL) is substantially higher than estimated with the currently available set of ClinVar and Leiden variants. An additional caveat to the predictive value of bioinformatic tools is that the determined pathogenicity of variants present in ClinVar and other clinical databases is often partially based on these tools, making the probability of a “pathogenic” score from these tools higher than by chance in many cases.

### Functional scores correlate with disease severity.

**Figure 4:**
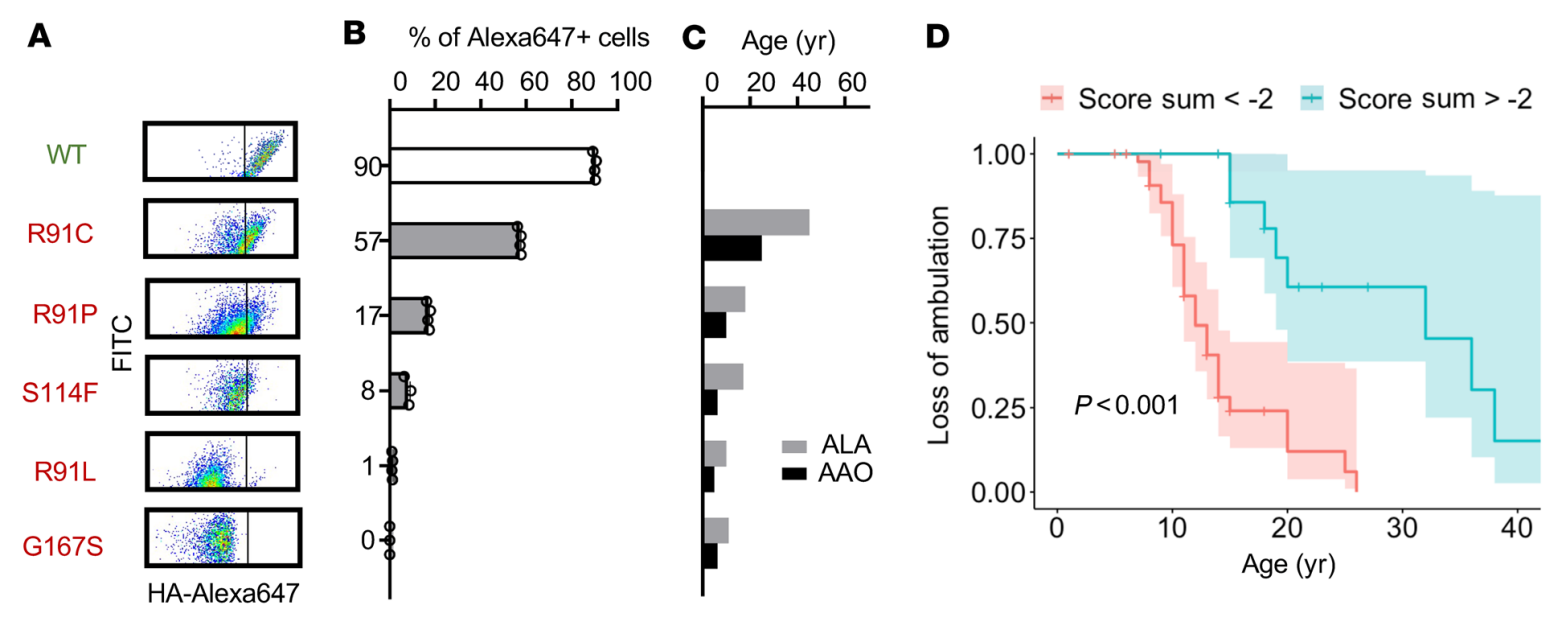
Relationship between disease severity and variant function. (**A**) FLOW cytometry dot plots showing the relationship between HA-immunofluorescence (HA-Alexa647) and YFP expression level (FITC, *y* axis) for ADG-HEK cells transduced with lentivirus to express either WT or mutant SGCB. (**B**) Quantification of the number of YFP-positive cells that also demonstrated positive HA cell surface staining. (**C**) Average age at onset (AAO) and age at loss of ambulation (ALA) for individuals homozygous for given variants in *SGCB*. (**D**) Cox’s proportional hazard curves for loss of ambulation among genetically diagnosed patients with LGMD with *SGCB* pathogenic variants with HA functional scores that sum less than –2 (severe) or more than –2 (milder).

There is a wide range of phenotypes for patients with LGMDR4/2E; age at symptom onset ranges from less than 1 to more than 20 years of age, some patients require a wheelchair at as early as 7 years of age, and some patients never require a wheelchair (13). This phenotypic variability is most strikingly exemplified with missense variants residing at position arginine 91 in SGCB. Three variants have been reported at this residue, with patients presenting with a mild phenotype if homozygous for an R91C, an intermediate phenotype if homozygous for R91P, and a more severe phenotype if homozygous for R91L variant. To validate this finding using our functional scores, we generated individual lentiviral constructs expressing YFP-SGCB-HA with the R91C, R91P, and R91L variants. The R91C, R91P, and R91L variants had diminished expression of 57%, 17%, and 1%, respectively (Figure 4, A and B), values that corresponded to the average age at onset and age at loss of ambulation for patients with these variants (Figure 4C). We further sought to determine if our measured functional score could be used as a measure of disease severity more generally and predict either age at onset or age at loss of ambulation (i.e., age at which the patient required a wheelchair) using all available clinical data for patients with missense variants (Supplemental Table 5). We performed a Cox’s proportional hazard analysis comparing the age at onset or age at loss of ambulation among patients with 2 severely nonfunctional missense *SGCB* alleles (functional score sum, <–2) with those with 2 fewer severe missense *SGCB* alleles (functional score sum, >–2). While there was no significant difference in the age at onset between these two groups, the age at loss of ambulation was significantly lower among patients with 2 severely nonfunctional SGCB alleles (Cox’s proportional hazard, *P* < 0.001) (Figure 4D), and there was a significant correlation between age at loss of ambulation and functional score sum (*r*^2^ = 0.22, *P* = 0.002), suggesting that the functional score derived here can not only predict pathogenicity but also disease severity.

### Functional constraint highlights structural features and protein-protein interactions.

**Figure 5:**
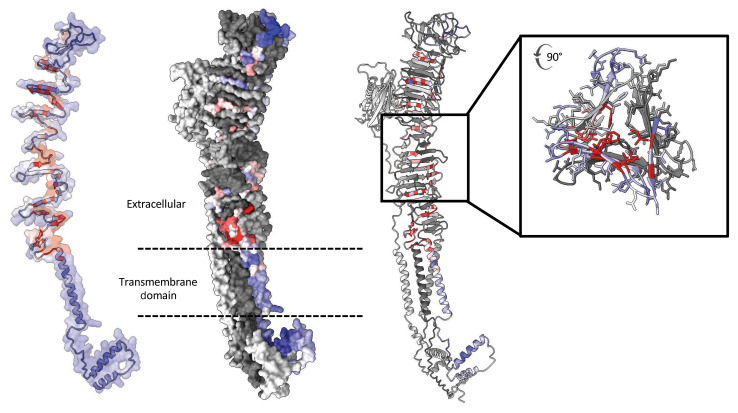
Structural insights from SGCB deep mutational scanning data. AlphaFold2 multimer model of SGCB-SGCA-SGCD-SGCG protein complex with SGCB surface colored, with average functional score of amino acid changes at each position and SGCA, SGCD, and SGCG colored in light gray. Ball-and-stick model of SGCB structure (modeled with SGCA, SGCD, and SGCG) highlighting the increased deleteriousness of amino acid changes at positions with side chains with multiple intermolecular interactions.

High-throughput functional screens, particularly those that test all possible amino acid changes, have the ability to inform our knowledge of the structure-function relationships present within a protein or protein complex. For example, as expected, we observed that amino acid changes that resulted in conservative changes (i.e., acidic to acidic amino acids) produced deleterious functional scores less often (101 of 1,171, 8.6%) than those that resulted in nonconservative changes (600 of 3,482, 17.2%; *P* < 4 × 10^–6^). A strong relationship between protein domain and functional score also was revealed when we compared the number of nonfunctional amino acid changes (functional score, <–0.5) in the cytoplasmic (54 of 1,282, 4.2%) or transmembrane domain (13 of 351, 3.7%) of *SGCB* with those in the extracellular topological domains of the protein (637 of 3,020, 21.1%; *P* = 7.6 × 10^–34^). Because the SGCB protein has not yet been crystalized, no structural models of SGCB or the sarcoglycan protein complex have been published to our knowledge. In order to further determine the relationship between functional scores and protein structure, we produced a model of SGCB protein structure using the multimer function of AlphaFold2 in the presence of SGCA, SGCD, and SGCG. The model produced was substantially different than the AlphaFold2 model produced using SGCB alone and revealed a structure in which SGCB, SGCD, and SGCG form a triple-helical quaternary protein structure with cobinding β sheets forming an interprotein β-barrel–like structure (Figure 5). We observed that the predicted structures form repeating regions of mutational intolerance corresponding to β sheet amino acids with inward-facing side chains harboring an excess of deleterious amino acid changes compared with outward-facing β sheet amino acids. Accordingly, the average number of interprotein contacts (<4 Å) among amino acid changes with functional scores of less than 0 was significantly greater than that with functional scores more than 0 for SGCB-SGCD (1.9 vs. 1.34, *P* < 2.4 × 10^–8^) and SGCB-SGCG (1.97 vs. 1.65, *P* < 9.3 × 10^–4^) interacting surfaces, the 3 proteins forming the triple-helical protein structure, and fewer for SGCB-SGCA interacting surfaces (0.28 vs. 0.71, *P* < 8.7 × 10^–4^). The proportion of sites with at least one interprotein contact between SGCB and either SGCD or SGCG for amino acid positions with reported pathogenic variants in SGCB was also significantly greater than that of sites without known pathogenic variants (100% vs. 60%, *P* = 3.7 × 10^–4^), further supporting the claim that intermolecular interactions are critical for SGCB function. Similar to previous deep mutational scans, we observed that amino acid changes resulting in proline were also significantly enriched for deleterious functional scores compared with other amino acid changes (40% vs. 24%, with scores <–0.5, *P* = 3.9 × 10^–14^). One region with strong mutational intolerance but unclear function is amino acids 90–99. Of particular note is position R91, which harbors 3 different known pathogenic variants (R91C, R91L, R91P). It may be that these positions on the cusp of the transmembrane domain (AA 60–90) are important for proper orientation of the protein complex in the membrane or are critical for initiating the helical structure of the 3 core SGC genes within the larger complex. It is unclear, however, why position R91 harbors more observed pathogenic variants than other sites in the SGCB protein.

### Inter-SGC protein interactions accurately predict SGCD and SGCG pathogenic mutations.

Because of the significant enrichment of damaging mutations at amino acids in SGCB that physically interact with SGCD and SGCG in the AlphaFold2 multimer protein structure model, we hypothesized that analogous changes at residues in SGCG and SGCD that interact with constrained SGCB residues would also lead to pathogenic changes. To test this, we aligned the protein sequence of SGCB to that of either SGCG or SGCD using Clustal Omega (23) and found that α helices and β sheets predicted by AlphaFold2 nearly perfectly aligned between SGCB and both SGCG and SGCD and that SGCD and SGCG are highly similar, with 53% of their amino acids being identical. We superimposed functional scores from SGCB onto the aligned amino acids in either SGCG or SGCD (i.e., L194S in SGCG corresponds with I218S in SGCB) determined from these protein alignments and found that nearly all clinically determined pathogenic variants have corresponding pathogenic scores in SGCB (range = –1.61 to 0.26) and all clinically determined benign variants have corresponding benign scores in SGCB (range = –0.46 to 0.47; Supplemental Figure 8 and Supplemental Table 6). Furthermore, pathogenic variants in SGCG or SGCD almost exclusively reside in β sheets (14 of 16 variants) involved in SGC-SGC contacts whereas nearly all benign variants reside within the intracellular or transmembrane domains of the proteins or in the extracellular domains of the proteins but outside of a β sheet (7 of 10 variants). These predictions of pathogenicity outperformed CADD, REVEL, and Polyphen scores (DMS AUC = 0.95, Supplemental Figure 8) in predicting pathogenicity of known variants in these genes. Together these findings suggest that DMS combined with structural information, either determined empirically or in silico, can inform pathogenicity predictions across proteins with similar structure and function.

### Insights into codon evolution from single amino acid saturation mutagenesis.

**Figure 6:**
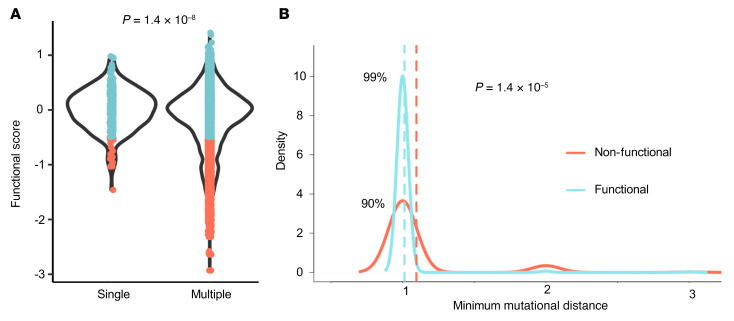
Trends in functional effect of variants based on codon use. (**A**) Distribution of HA functional scores for amino acid changes possible with a single nucleotide change versus those possible only with multinucleotide changes. (**B**) Density plot depicting the minimum number of nucleotide changes required for each amino acid to be changed either to a nonfunctional (score, <–0.5) amino acid (red) or a different functional (score, >–0.5) amino acid (blue). *T* tests were used to test for differences in distribution between groups.

Unlike single nucleotide mutagenesis, which enables the measurement of all possible variants generally accessible during evolution, single amino acid saturation mutagenesis enables measurement of amino acid changes not easily accessible by evolution, i.e., codon changes requiring 2–3 nucleotide changes. We aimed to determine if amino acid changes that resulted from single nucleotide changes were more or less likely to lead to a nonfunctional protein compared with amino acid changes that are only possible following 2–3 adjacent nucleotide changes, consistent with the well-established finding that more similar codons tend to encode more biochemically similar amino acids (24, 25). While we observed no difference between the average functional score of amino acid changes caused by single nucleotide changes and those same amino acid changes resulting from multinucleotide changes (*t* test, *P >* 0.05), we observed a strong enrichment of deleterious functional scores among amino acid changes only possible from multinucleotide changes compared with amino acid changes possible from single nucleotide changes (score, –0.18 vs. 0.01, *t* test, *P* = 1.4 × 10^–8^; Figure 6A) and the average mutational distance of deleterious amino acid changes was significantly greater than that of neutral amino acid changes (2.4 vs. 2.2 nucleotide changes, *P* = 1 × 10^–58^; Figure 6B). This observation was also true in a recently published DMS of MSH2 (26), where 7% of amino acid changes reachable by a single nucleotide change resulted in a nonfunctional protein (MSH2 functional score, >0) compared with 12% of amino acid changes possible only by more than 1 nucleotide change that resulted in nonfunctional protein (*t* test, *P* < 5 × 10^–21^). To test more specifically that specific codons were selected by evolution to be maximally distant from deleterious amino acid changes, we calculated the average distance of possible codons at each position that encode the WT amino acid and compared it with the observed distance of the codon chosen by evolution. We observed that the difference between the utilized codon and the average of possible codons for functionally deleterious amino acid changes was significantly greater than for those that were functionally neutral (+0.029 mutational distance, paired *t* test, *P* = 8 × 10^–6^), whereas there was no significant difference for neutral amino acid changes (–0.004 mutational distance, paired *t* test, *P* = 0.26). Finally, when we restricted our analysis to only those amino acid changes possible by 1 nucleotide change, we saw that deleterious amino acid changes were more often encoded by a codon that requires a greater number of nucleotide changes to obtain the amino acid change compared with neutral amino acid changes (82% vs. 70%, *P* = 1.4 × 10^-13^), effectively maximizing the mutational distance from the current amino acid to the array of possible deleterious amino acids by codon choice.

## Discussion

The sarcoglycan genes and *SGCB* in particular are among the most frequently mutated genes underlying LGMD (10, 17). Like many recessive disease genes, affected patients carrying biallelic loss-of-function variants (i.e., premature termination codons or indels resulting in frame shifts) provide strong evidence for variant pathogenicity. However, this evidence is lacking for the majority of missense variants observed in patients. Immunofluorescence based cellular assays of sarcoglycan function provide the necessary evidence (ACMG criteria PS3) to aid in the interpretation of observed and yet unobserved genetic variation. In this study, we performed and integrated massively parallel assays of SGCB function, SGCB cell surface expression and SGCA cell surface expression, and generated a near-complete map of the functional effect of missense variants in the LGMD gene *SGCB*.

Measured functional scores for variants are highly accurate in predicting the pathogenicity of known disease-causing variants, outperforming the newest prediction algorithms. This is the first time to our knowledge that a full-length muscular dystrophy gene has been subjected to DMS, suggesting that pooled functional screens are a viable method of large-scale functional assessment of protein-coding genetic variation in other muscular dystrophy genes (*SGCA*, *SGCD*, *SGCG*, etc.), particularly those whose cell surface expression is key to their function.

Nearly all known pathogenic variants in *SGCB* that have been evaluated in primary tissue samples from patients show strong effects on the cell surface expression of not only SGCB but other members of the dystrophin-associated protein complex, particularly the other 3 sarcoglycan proteins, SGCA, SGCD, and SGCG (14, 15). Accordingly, our functional measurements were highly consistent with expert-reviewed variant classification records from the ClinVar database and the Leiden genetic variant database that often use sarcolemmal expression in patient muscle tissue as their functional evidence. Our goal is to provide functionally relevant evidence to be used in variant resolution, which would satisfy the requirements set forth by the ACMG to add strong evidence for the pathogenicity of variants with negative functional scores in our assays (PS3 criteria) (27, 28). Although the functional scores defined here were perfectly accurate in predicting the pathogenicity of variants with strong evidence in ClinVar, one variant (E28G) was listed in the Leiden SGCB database as “benign” without any cited evidence. Our assay results would suggest that this variant is mildly deleterious (functional score, –0.33) but not below our cutoff of –0.5 for classifying a variants as nonfunctional despite being in the less constrained cytoplasmic domain of SGCB. The functional effect maps we present may, therefore, allow for the potential reclassification of variants with limited evidence present in clinical databases.

We found that our measured functional scores were a better predictor of known pathogenic and benign variants than Polyphen2, CADD, or REVEL scores. Although the predictive value of REVEL was high using the relatively small number of known pathogenic/benign variants, we estimate that true false positive rate for REVEL to be substantially higher when a larger number of confirmed disease-causing variants are defined. If using conservative cutoffs for a benign function score of more than 0.5 and a deleterious REVEL score of more than 0.75, 420 of 1,870 or 22% of single-nucleotide variants would be considered false positives (i.e., deleterious by REVEL and benign by DMS score), whereas only 1 known benign variant lies within this range.

Our findings that disease severity, as measured by age at loss of ambulation, are related to functional score in our assay suggests that cell surface expression of SGCB and the SGC protein complex is potentially a quantitative trait that determines, in part, the stability of the sarcolemma and the integrity of muscle cells over the course of a lifetime. In the future, it will be interesting to determine if functional scores measured here correlate with more subtle muscle traits, such as sarcopenia or exercise intolerance in the general population, at least among individuals with protein-altering genetic variation in these genes.

The pattern of deleterious amino acid changes across the SGCB gene closely mirrored the predicted protein structure produced using AlphaFold2 (29). The co-occurrence of deleterious amino acid changes within predicted β sheets highlighted the importance of interprotein interactions, particularly between SGCB and SGCD, in producing a functional protein complex capable of being assembled and transported to the cell membrane. Intriguingly, there was minimal effect when amino acids within the intracellular domain or transmembrane domain of SGCB were changed. Furthermore, the importance of amino acid 91 is not yet clear, although it may be that its position at the transition between the transmembrane domain and the extracellular domain is critical for some yet undetermined function. Overall, we believe that our scores and their pattern corroborate the predicted AlphaFold2 structure and improve our understanding of the domains and interactions important for SGCB function. This was further demonstrated by our ability to predict the pathogenicity of SGCD and SGCG variants with high accuracy using SGCB functional scores and knowledge of the structural relationship between the 3 proteins. By aligning the 3 genes’ protein structures and superimposing SGCB scores on SGCD and SGCG, we were able to gain further insight into the importance of interprotein contacts by accurately predicting pathogenic variants in these two related proteins. Only one known pathogenic variant in either SGCG or SGCD (C283Y in SGCG) was predicted to be benign as it aligned to SGCB position C307Y (functional score, 0.26). The site is within the C-terminus of the protein, not within a β sheet, and has limited contacts with other SGC proteins (4 contacts with SGCG and 0 with the other 2 SGC proteins). The mechanism of functional disruption of this variant, if it is truly pathogenic, remains unclear. There may be other proteins that the amino acid at this position interact with that affect SGCG function but do not alter SGCB or SGCA trafficking. However, direct functional screening of all variants in both SGCD and SGCG will be required to confirm these predictions.

Overall, SGCB is moderately tolerant of protein-altering genetic variation, with 16% of single-nucleotide missense variants demonstrating nonfunctional scores (score, <–0.5). This number jumps to 30% when considering all possible amino acid changes, however, implying that evolutionary forces lead to the selection of specific amino acids and even specific codons with fewer nonfunctional alleles reachable by single nucleotide changes. We believe this is the first time this phenomenon has been reported in DMS data and that this helps to explain the relatively low rate of pathogenic variants observed in human populations despite the high degree of evolutionary conservation across species. These data suggest that proteins may have evolved a buffer against deleterious protein alterations by selecting amino acids and codons with a lower probability of producing nonfunctional proteins.

The bimodal distribution of functional scores is similar to previous deep-mutational scanning reports, with most *SGCB* missense variants demonstrating either a clear nonfunctional score or a neutral score. It is yet unknown, however, whether functional scores between the means of these functional classes represent true quantitative measures of protein function or experimental noise. For example, there are 13 VUSs with functional scores between those of the highest scoring known pathogenic variant (R91C, score, –0.72) and the lowest scoring known benign variant (E28G, score, –0.33). No known pathogenic or benign variants exist within these borderline functional scores; however, this may be due to sampling bias with only those patients with severe phenotypes and clear genetic findings entered into clinical databases. When more clinical data from patients with a wider range of disease severity are investigated in the context of these functional scores, we will likely identify individuals with more intermediate phenotypes and correspondingly intermediate functional scores. For variants that appear to act as hypomorphs in functional assays, it is unclear whether these are likely to be considered pathogenic in the strictest sense or rather should be considered factors contributing to a spectrum of limb-girdle muscle weakness present in the general population. The relationship we observe between functional score and age at loss of ambulation, even among patients with 2 alleles in the pathogenic range of functional scores, suggests that SGCB function is not purely normal or loss of function but a quantitative trait captured at least in part by measuring its ability to complex with other SGC genes and be transported to the cell surface as our assay measures. As large data sets of nonsyndromic individuals are sequenced and integrated with clinical information and physiological measurements, it may be possible to correlate more directly the role of *SGCB* genetic variation in the range of normal muscle function in addition to disease risk as explored here.

A limitation of our experimental design is the inability to measure the effects of variants on endogenous mRNA or protein levels, the effect of both coding and noncoding genetic variation on splicing, and the effects of structural variation (both small indels and large CNVs). In ClinVar, there are 11 known pathogenic splicing mutations. In the future, splicing effects could be measured by mutagenizing splice regions in the endogenous locus and measuring cell surface expression of the SGC complex in primary muscle cells. It is clear that both small and large DNA insertions or deletions can disrupt SGCB function, with frame shift variants having a high likelihood of being pathogenic. Whole-exon deletions and in-frame indels are harder to predict, however, and would likely require functional assessment as we performed here. However, these are highly unique variants in terms of length, position, and composition, and, thus, they are better suited to individual testing if they are observed in an individual. Another potential limitation is that our assay does not measure muscle integrity or atrophy over time directly and would not detect defects in interactions between SGCB and other proteins in the membrane or extracellular space that are important for muscle function. Our results may be further improved through the use of orthogonal functional assays or specialized assays for protein function.

Here, we demonstrate that high-throughput functional assays can accurately measure the effect of protein-coding genetic variation in the LGMD gene *SGCB*. The map of functional effects we present has the ability to improve classification of variants observed in patients with LGMD and aid our understanding of the structure of an important member of the dystrophin-associated protein complex. When used together with available lines of evidence, these results will add confidence to variant interpretation and potentially allow patients with pathogenic variants to be treated with gene-specific therapeutics for which they would have otherwise been ineligible. Future work will explore these methods for additional SGC and muscular dystrophy genes with similar pathological mechanisms.

## Methods

### Plasmid constructs.

The full-length cDNA of human WT *SGCA*, *SGCD*, and *SGCG* was synthesized and individually cloned into a lentiviral vector by Genewiz. A YFP-*SGCB* construct was also synthesized and cloned into the same lentiviral vector expressing WT human β-sarcoglycan followed by YFP. A HA tag was further added to the C-terminal end of the *SGCB* by site-directed insertion mutagenesis (Takara). The new construct was termed YFP-*SGCB*-HA. Single or double variants in β-sarcoglycan were introduced by site-directed mutagenesis according to the manufacturer’s protocol (Takara). All constructs were verified by Sanger sequencing (Genewiz).

### Cell culture and generation of ADG stable cell line.

HEK293 (ATCC, CRL-1573) were cultured in DMEM (high glucose, MilliporeSigma), supplemented with 10% fetal bovine serum (Atlanta Biologicals). Cells were maintained in a humidified atmosphere of 5% CO_2_ at 37°C. During routine passaging, cells were washed with PBS and dissociated with trypsin-EDTA 0.05%. HEK293 cells were tested for endogenous α-, β-, γ-, and δ-sarcoglycan by Western blot assay; all were undetectable. To generate monoclonal stable cell lines coexpressing α-, γ-, and δ-sarcoglycan, HEK293 cells were tri-transduced with lentiviruses containing WT SGCA, SGCD, and SGCG as described above. Colonies formed by expansion of single cells were screened by PCR and Western blot for the presence of human sarcoglycans (Supplemental Figure 2A). Clone 10, expressing α-, γ-, and δ-sarcoglycan at about 1:1:1 stoichiometry, was used in the described experiments and deemed the ADG-HEK cell line. Protein expression of all 4 SGC proteins was confirmed after transduction of ADG-HEK cells with WT SGCB lentivirus by Western blot (Supplemental Figure 2B).

### Western blotting and immunocytochemistry on nonpermeabilized cells.

Cells were lysed by the addition of RIPA buffer (50 mM Tris-HCl, pH 7.4, 150 mM NaCl, 1% NP-40, 0.25% Na-deoxycholate, and 1 mM EDTA) and protease inhibitor cocktail (MilliporeSigma). Protein concentrations of lysates were determined using the BCA Protein Assay Kit (Thermo Fisher Scientific). Equal amounts of protein were run on a 10% gel and transferred to a nitrocellulose membrane. The blots were blocked with 5% nonfat dry milk in PBS for 1 hour and probed with anti–α-sarcoglycan (Santa Cruz, sc-390647/F-7), β-sarcoglycan (Santa Cruz, sc-393679/F-6), γ-sarcoglycan (Leica, NCL-d-SARC/Ad1/20A6), and δ-sarcoglycan (Leica, NCL-g-SARC/35DAG/21B5) at 4°C overnight. Secondary antibodies included horseradish peroxidase–labeled anti-mouse IgG (Cell Signaling, 7076). Blots were developed with ECL (GE Healthcare). Images were taken using the G: Box Chemi XT4, Genesys (version 1.1.2.0, Syngene). For immunocytochemistry, cells were fixed using 4% paraformaldehyde but not permeabilized. These cells were then stained for cell surface expression using an anti-HA antibody (Biolegend, 682404/16B12) followed by staining with an Alexa Fluor 647 goat anti-mouse secondary antibody (Thermo Fisher Scientific, A-21236), an Alexa Fluor 647–conjugated anti-HA antibody (Biolegend, 682404/16B12), a Pacific Blue anti-HA antibody (Biolegend, 901525/16B12), or an Alexa Fluor 647–conjugated anti-α-sarcoglycan antibody (Santa Cruz, sc-390647 AF647/F-7). Images were taken using a fluorescence microscope (Nikon 80i).

### Mutation library construction.

To achieve all possible amino acid substitutions of the entire SGCB (954 bp, 318 amino acid residues), the complete library was divided into 6 sublibraries (Supplemental Figure 9), and each contained a different 144–162 bp fragment of the SGCB gene (SGCB-A, residues 1–54; SGCB-B, 55–108; SGCB-C, 109–162; SGCB-D, 163–216; SGCB-E, 217–270; and SGCB-F, 271–318). Each sublibrary was custom synthesized by Integrated DNA Technologies as a pool of oligonucleotides such that each oligonucleotide encoded an “NNN” at one amino acid position but was otherwise homologous to the template (Supplemental Table 1). To enable initial PCR amplification and insertion of sublibraries into plasmids by Gibson assembly (described below), WT SGCB homologues sequences (>20 bp long) were added at both ends of the sublibraries.

For cloning of each sublibrary into lentiviral vector YFP-SGCB-HA (described above), the synthesized oligonucleotide pools were dissolved and diluted at 1:100 and used as template for PCR amplification with CloneAmp HiFi Polymerase (25 cycles, Takara). The product was purified using the Nucleospin Gel and PCR Clean-up kit (Takara). Backbone plasmid YFP-SGCB-HA was PCR amplified with primers that are complementary to the WT SGCB homologues sequences at the ends of each sublibrary. The resulting linearized backbone that carried the remaining template sequence was digested with DpnI to deplete starting plasmid, separated from nonspecific fragments by electrophoresis on a 1% agarose gel, and purified using the gel clean-up kit (Nucleospin). Each sublibrary was inserted into YFP-SGCB-HA plasmids via Gibson assembly (NEBuilder HiFi DNA assembly Master Mix) according to the manufacture’s protocol, so that it replaces the equivalent part of the WT SGCB cDNA sequence. The assembly reactions were purified and transformed into E. cloni DUO Electrocompetent cells (Lucigen) with 5% of cells plated to estimate cloning efficiency and 95% of cells grown in liquid media for 12–16 hours at 37°C in LB supplemented with 100 mg/mL ampicillin. We obtained an estimated >40,000 colonies per section to ensure >10× colonies per potential sequence present within each library. Each library was isolated using an Endo-free Maxiprep kit (Qiagen). Library diversity was assessed by sequencing on an Illumina MiSeq 2 × 250 bp lane such that the entire mutated section was sequenced in both directions and a portion of the nonmutagenized section was sequenced as an internal control for background mutations.

### Lentiviral preparation and transduction.

SGCB cDNA subsection libraries (A–F) were packaged into lentivirus at the Hope Center Viral Core facility at Washington University by cotransfecting HEK293T cells (ATCC) with the transfer plasmid pool plus envelope and packaging vectors (pMD2.G, Addgene 12259 and psPAX2, 12260). For each pool, 4 × 10^6^ cells were plated onto each of six 100 mm dishes and transfected with 10 μg total plasmid (2.0:1.0:1.3 transfer/envelope/packaging ratio), using Lipofectamine 3000 (Thermo Fisher Scientific). Media were replaced after 6 hours, and viral supernatants were collected at 24 hours, filtered using a 0.45 mm filter (Millipore), and stored at –80°C. Viral titer was determined by qPCR and empirically estimated with a dilution series of transduced cells followed by FACS to measure the proportion of YFP-positive cells. For each biological replicate for each section, ADG-HEK cells were transduced with mutant library at low multiplicity of infection (<0.1), by applying 1.5 mL viral supernatant (with 8 mg/mL polybrene) to a 100 mm dish (TPP) containing approximately 5 × 10^6^ cells, with three 100 mm dishes per replicate to maintain library diversity. After 72 hours, transduced cells were bulk sorted for the presence of YFP using a FACS Aria II (BD) into DMEM containing 100 U/mL penicillin and 100 g/mL streptomycin. Cells were grown for 5–7 days and then stained for cell surface expression of YFP-SGCB-HA or SGCA.

### Functional selection.

Three measurements of function were obtained for each mutagenized section of SGCB. First, transduced cells were harvested during passaging to obtain a measurement of the frequency of each mutation present in the unselected pool of transduced cells. A sample of transduced cells after selection for YFP expression was obtained to measure the abundance of each mutation in YFP-positive cells compared with all transduced cells to measure the effect, if any, on total protein level. Third, YFP-positive sorted cells were further labeled with Alexa Fluor 647–conjugated antibody for FACS. Briefly, live cells were collected using trypsin-EDTA 0.05%. Thereafter, cells were blocked in blocking buffer (0.5% BSA-PBS) for 20 minutes on ice; half the cells were incubated with Alexa Fluor 647–conjugated anti-HA, and the other half were stained with Alexa Fluor 647–conjugated anti–α-sarcoglycan antibody on ice for 30 minutes. After incubation, cells were washed twice with PBS. The cells were fixed with 2% paraformaldehyde on ice for 20 minutes. Next, cells were resuspended in ClearFlow Sheath Fluid (Leinco Technologies) and sorted using FACS Aria II into bins according to the intensity of Alexa Fluor 647 signal. After the single cells were selected using forward and side scatter, a histogram of the FITC was created to select for YFP-positive cells. For those YFP-positive cells, a histogram of the Alexa Fluor 647 was further created and gates were drawn across the library based on red intensity: low (bin 1, 5%), medium low (bin 2, 5%), medium high (bin 3, 5%), and high (bin 4, 5%). Cells harboring single or double variants in β-sarcoglycan were labeled with Alexa Fluor 647–conjugated antibody in the same ways as described above. The single cells were gated and analyzed for YFP expression. Furthermore, the YFP-positive population was analyzed for the expression of Alexa Fluor 647 by MACS Flow Cytometer (Miltenyi Biotec). The data analysis was performed using FlowJo software (TreeStar Inc). Each assay was performed in duplicate starting from transduction.

### Amplification and sequencing of integrated SGCB.

After selections, genomic DNA was isolated from more than 500,000 cells using a DNeasy Blood and Tissue DNA extraction kit (Qiagen) and sequenced for the mutated region. For each sample, 16 replicate PCR reactions were performed and pooled, each with 200 ng genomic DNA as template. Mutated sections from integrated inserts were PCR amplified with NEBnext High-Fidelity polymerase (NEB). Each set of 16 replicate amplicons was pooled and purified with SPRI beads. Mutated sections were then amplified for 15 cycles using primers with 5′ overhangs of Illumina adapter handles and then secondarily amplified for 25 cycles using dual-indexed illumina adapters. The resulting Illumina libraries contained inserts ranging from 200 to 240 bp, enabling full read coverage in both directions for 2 × 250 bp sequencing and more than 50 bp overlap for 2 × 150 bp sequencing. Illumina libraries were either sequenced using 2 × 250 bp flow cells on a MiSeq or 2 × 150 bp flow cells on a NovaSeq.

### Variant enrichment, function scores, and pathogenicity predictions.

Within each sample, raw read counts for each mutant codon were counted and per-mutation frequencies were calculated; any variant read counts under 50 per 1 million reads across the 4 sorted bins were removed from further analysis. For both HA-SGCB and SGCA, functional scores were taken as the log_10_ ratio of the frequency in bin 4 (high expression) over that in bin 1 (low expression) and then normalized across chunks such that the functional scores for synonymous mutations in each chunk have an average of 0 and a standard deviation of 0.5. For amino acid substitutions represented by more than one equivalent codon substitution, the median was taken across those codons’ functional scores to yield an amino acid–level score. The median score was taken for each amino acid functional score across biological replicates.

### Clinical and population variants.

SGCB missense variant classifications were obtained from ClinVar on September 6, 2022; the Leiden database; or extracted from the literature (Supplemental Table 2). If a variant was called pathogenic in at least one source and never called benign or likely benign, we considered it pathogenic. Likely pathogenic variants were those only called likely pathogenic. Variants were called “conflicting” if the variant was reported both as benign or likely benign and also a VUS or pathogenic. Benign variants were defined as those listed as benign in at least one source or observed in the homozygous state in more than 1 individual in a population database. Population variation was obtained from the gnomAD database (30), combining whole-genome and -exome calls from versions 2.1.1 and 3.0. Population variant was also obtained from the UKBB exome sequenced data set (*n* = 500,000).

### Bioinformatic prediction scores.

All *SGCB* missense variants were scored by REVEL (22), CADD (31), and PolyPhen-2 (32). Scores were available only for missense variants reachable by single-base variants (SNVs); for amino acid substitutions that could arise from more than 1 SNV, the mean of those SNVs’ scores was taken. These scores were only used for comparison to our results from in vitro functional experiments and not used in the derivation of functional scores. Functional scores are entirely independent of these in silico predictions.

### SGC gene structure and conservation.

The SGCB predicted structure was generated using AlphaFold2 (29) using the multimer algorithm, including the SGCB, SGCA, SGCD, and SGCG protein sequences. The predicted protein structure of SGCB in the context of the other 3 SGC proteins was rendered with ChimeraX (33).

### Statistics.

Functional scores were defined as the log ratio of mutant read counts in cells with high cell surface SGC protein level and cells with low cell surface SGC protein level. For all quantitative measurements, mean ± SEM are reported. Normal distribution of the sample sets was determined before application of 2-tailed Student’s *t* test. A 2-sample Student’s *t* test was used to compare the differences between 2 groups. Kaplan-Meier method was used to estimate the probability of survival, and the log-rank test was used to compare the overall survival difference between groups. Correlation between variables and significance was determined using linear regression with the lm function in R. All statistical tests were 2 sided, and the differences were considered significant when *P* was less than 0.05.

### Study approval.

Collection of clinical information from the Institutional Review Boards of Washington University School of Medicine, Fundación Canaria Instituto de Investigación Sanitaria de Canarias, and John Walton Muscular Dystrophy Research Center. Written informed consent was obtained from all participants for participation in these studies.

## Author contributions

GH and CCW designed research studies. CL, JW, SP, and JD conducted experiments. GH provided statistics analysis. JAP and JDM provided clinical data. CL, GH, and CCW wrote the manuscript. CL, GH and CCW analyzed data. CL, JW, SP, JD, GH, CCW, JAP, and JDM revised the manuscript

## Supplementary Material

Supplemental data

Supplemental tables 1-6

Supplemental video 1

## Version 1 06/15/2023

Electronic publication
