# Supplementary material for: Comprehensive functional characterization of SGCB coding variants predicts pathogenicity in limb-girdle muscular dystrophy type R4/2E
Source: J Clin Invest. 2023 Jun 15;133(12):e168156. doi: 10.1172/JCI168156 (PMC10266784; doi:10.1172/JCI168156)
Supplement: Supplemental data [file jci-133-168156-s147.pdf]

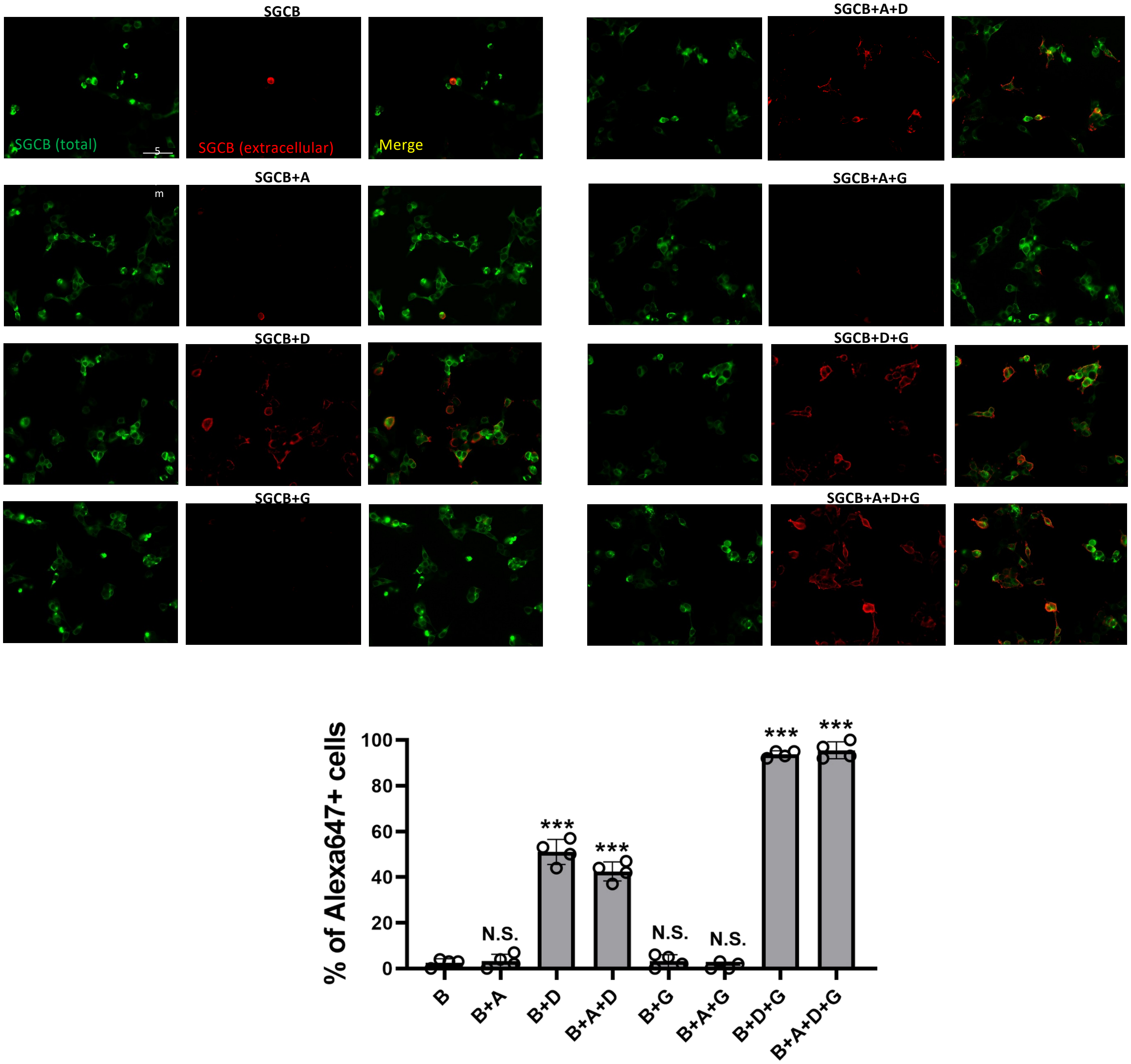


**Figure S1. Cell surface SGCB expression in the context of other SGC genes.** HEK cells were transduced with various combinations of SGC genes via lentivirus and then measured for total (green) and cell-surface (red) SGCB expression. Co-expression with all three other SGC genes (bottom right) demonstrated the most robust cell-surface SGCB expression and was used for all subsequent experiments.

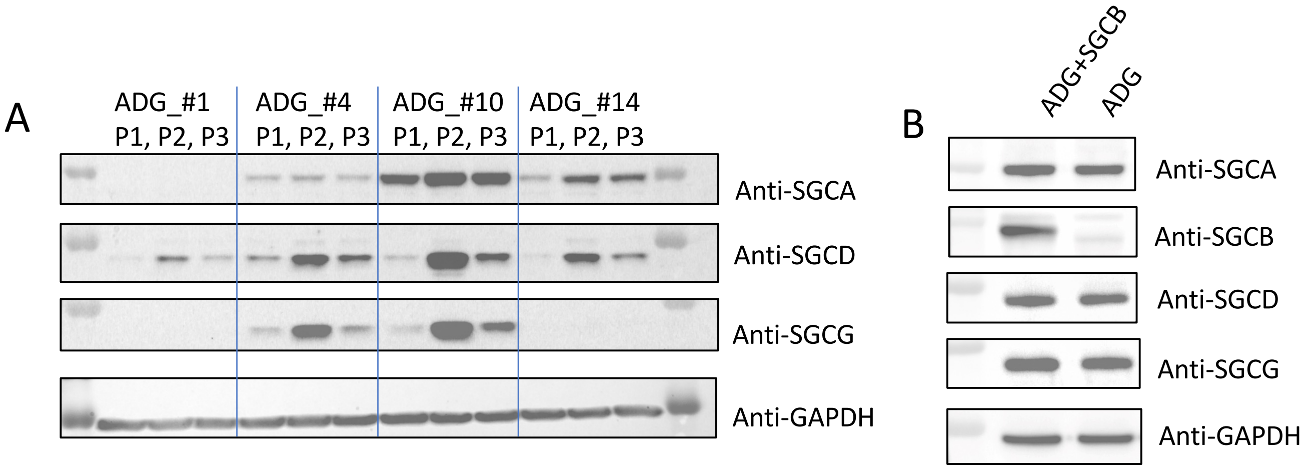


**Figure S2. Western Blots of Stable ADG-HEK cells.** A) HEK293 cells were transduced with three lentiviruses expressing SGCA, SGCD and SGCG, respectively. Cells were single-cell sorted using FACS and grown to generate clonal cells lines. Cell lines positive for lentiviral DNA from each gene (1, 4, 10, 14) were screened for protein expression by western blot at three different passages after transduction (P1, P2 and P3). ADG-HEK line #10 was used for all DMS experiments. B) ADG-HEK cells (line #10, passage #5) were transduced with SGCB wild-type lentivirus and assessed for SGCA, SGCB, SGCD and SGCG protein level by Western blot.

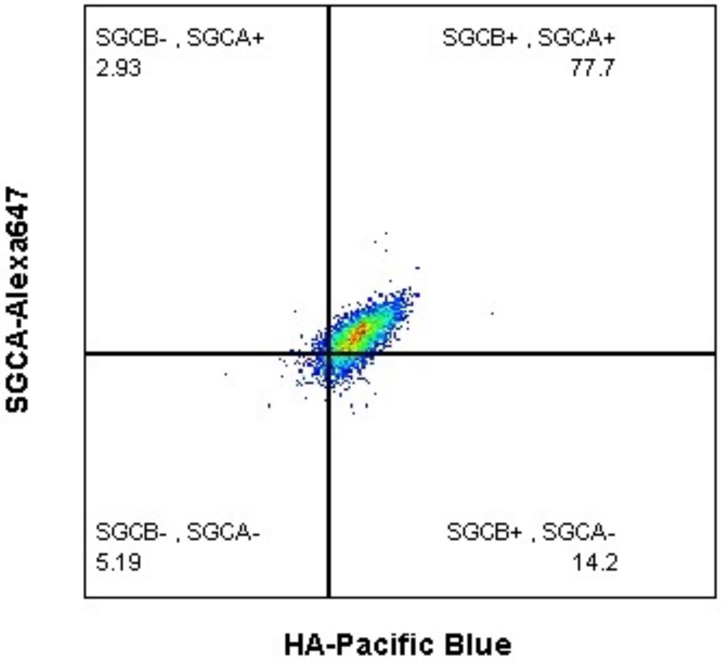


**Figure S3. Flow cytometry of ADG-HEK cells transduced with SGCB-WT and co-stained for cell surface SGCB and SGCA.** YFP positive cells were firstly gated to exclude non-transduced cells, and then quadrant gates were drawn based on negative control cells (i.e. SGCB-WT transduced cells without antibody staining, or stained with HA-Pacific blue or SGCA-Alexa647), 77.7% of cells were positive for both SGCB and SGCA cell surface protein. There was a strong positive correlation between the amount of SGCB cell surface protein and SGCA cell surface protein. A larger proportion of cells were SGCB positive (91.9%) than were SGCA positive (80.63%). This may simply be due to differences in background fluorescence for the two antibodies and the requisite cutoffs for positivity as a result.

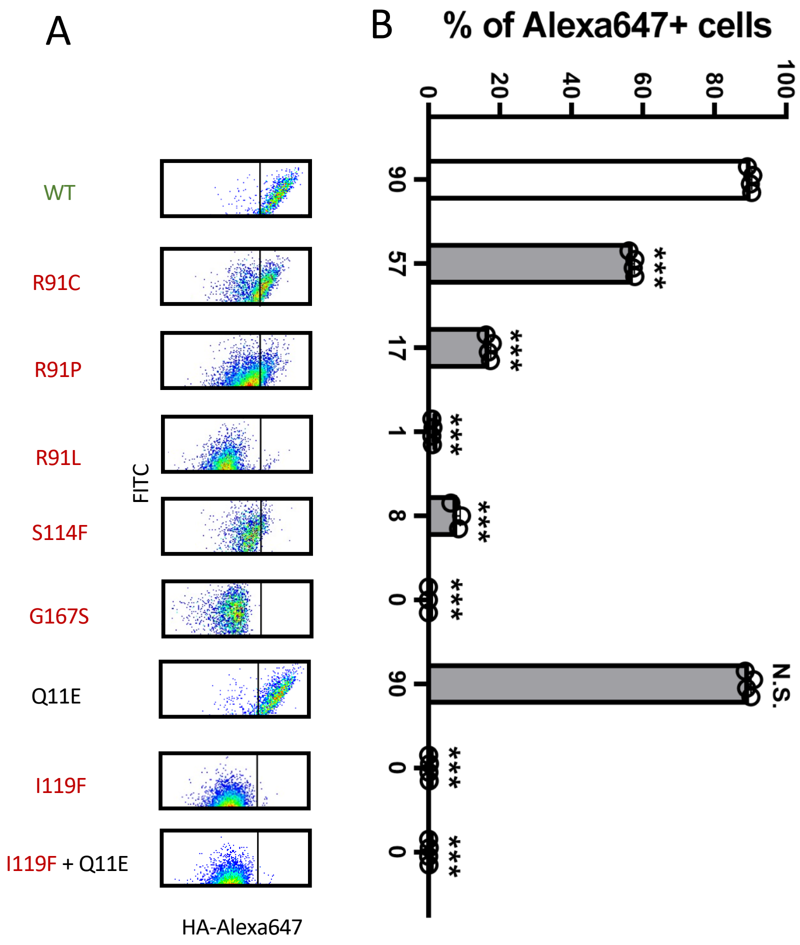


**Figure S4. Flow cytometry of HA-SGCB cell-surface immuno-stained ADG-HEK cells transduced with mutation containing SGCB.** (A) FLOW cytometry dot plots showing the relationship between HA-immunofluorescence (HA-Alexa647) and YFP expression level (FITC, y-axis) for ADG-HEK cells transduced with lentivirus to express either wildtype (WT) or mutant SGCB. B) Quantification of the number of YFP-positive cells that also demonstrated positive HA cell-surface staining. ***p<0.001 compared to WT. Disclosure: This figure contains data present in Figure 4 in the main text.

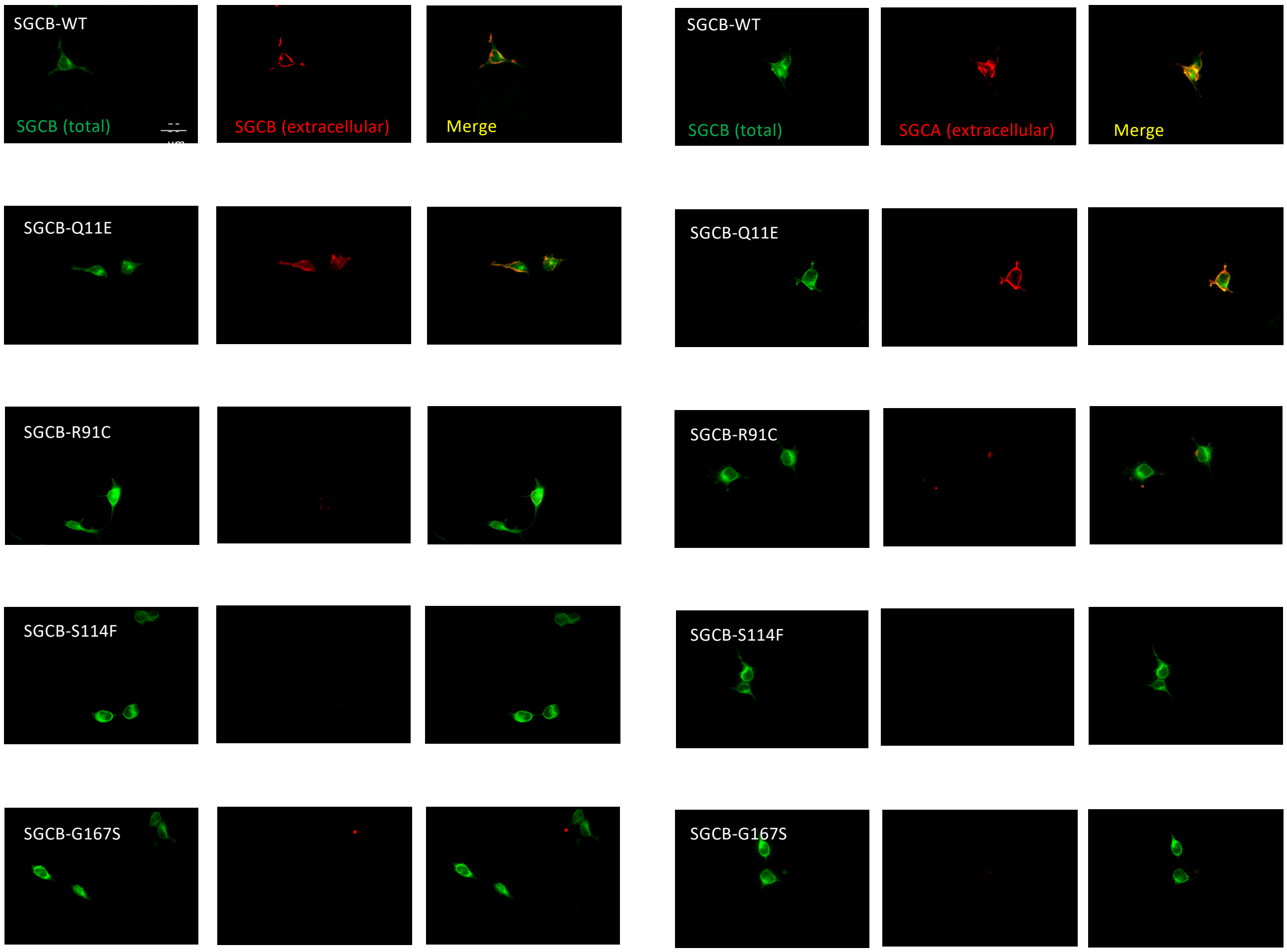


**Figure S5. Cell surface expression of SGCB and SGCA for single SGCB variants.** ADG-HEK cells were transduced with single SGCB variants via lentivirus and then measured for total SGCB (green) and either cell-surface SGCB or cell-surface SGCA expression (red).

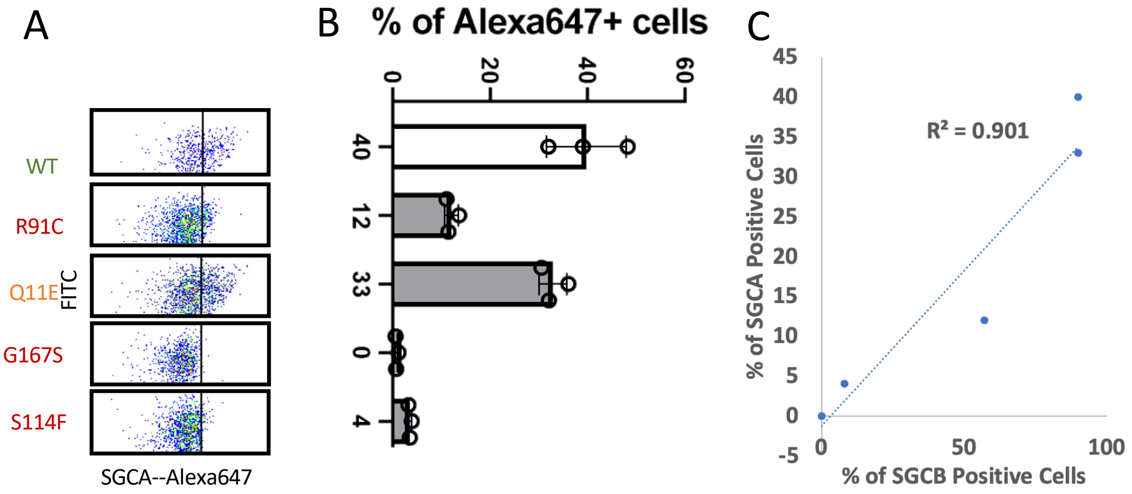


**Figure S6. Flow cytometry of SGCA cell-surface immuno-stained ADG-HEK cells transduced with mutation containing SGCB.** A) FLOW cytometry dot plots showing the relationship between SGCA-immunofluorescence and YFP expression level (FITC, y-axis) for ADG-HEK cells transduced with lentivirus to express either wildtype (WT) or mutant SGCB. B) Quantification of the number of YFP-positive cells that also demonstrated positive SGCA cell-surface staining. C) Correlation between percent positive cells for single variant transduced cells for cell-surface SGCB protein and cell-surface SGCA protein.

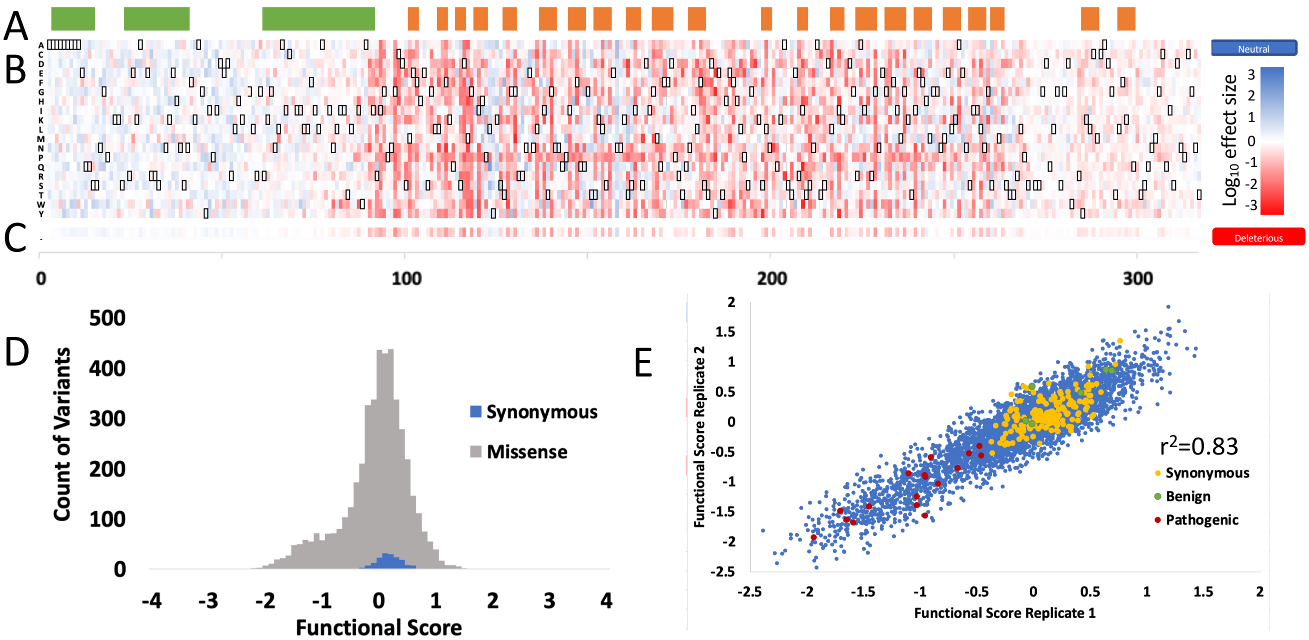


**Figure S7. Functional Effect Map of SGCA.** A) Alpha helices (green) and beta-sheets (orange) as predicted from the AlphaFold multimer model of SGCB modelled with SGCA, SGCG and SGCD. B) SGCA functional score biological replicate median values are displayed as a heatmap. Scores range from damaging (red) to benign variants (blue). Missing or low confidence data are shown in yellow. Synonymous changes are bounded in black boxes. C) Average SGCA functional score per position. D) Histogram of SGCA functional scores demonstrate a bimodal distribution with synonymous variants (blue) showing a narrow range of scores around 1 (i.e. enriched in SGCA bin 4). E) Correlation between biological replicates of SGCA-stained ADG-HEK cells transduced with SGCB libraries (libraries A-F are included) with ClinVar pathogenic variants (red) and benign variants (orange) highlighted.

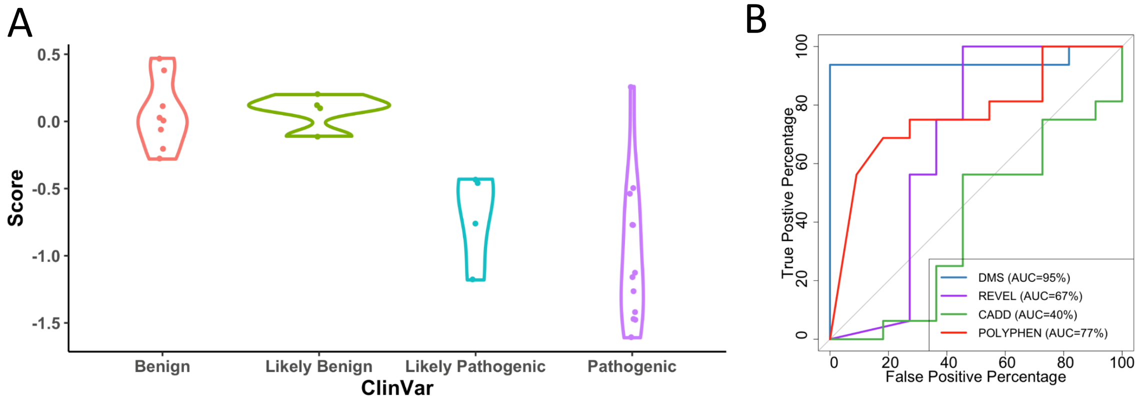


**Figure S8. Concordance of predicted functional scores in SGCD and SGCG with existing clinical classifications and computational predictions of pathogenicity.** A) Predicted functional scores (y axes) for patient missense variants from ClinVar/Leiden databases by variant classification (x axes). B) Receiver Operator Curves (ROC) predicting Pathogenic or Benign classification (27 variants) for the functional score predicted by alignment to SGCB (DMS), REVEL, CADD or PolyPhen.

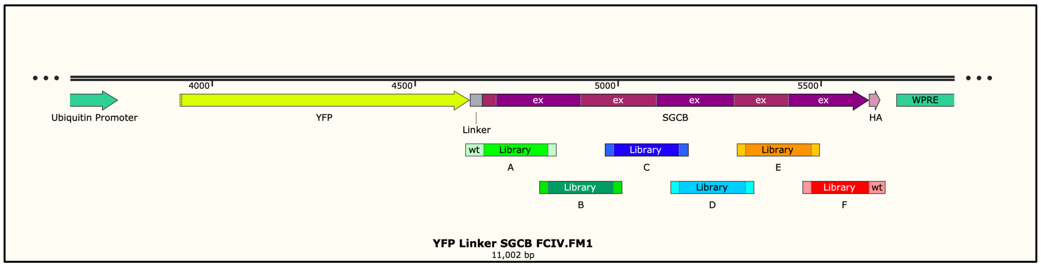


**Figure S9. Diagram of SGCB sub-libraries.** Libraries of oligos, each containing a single codon encoded as 'NNN' were purchased from IDT and named pools A-F and covered the entirety of the SGCB coding sequence with 42bp of overlapping sequence between each pair of sub-library.
